# Molecular and Clinical Aspects of Chronic Manifestations in Chagas Disease: A State-of-the-Art Review

**DOI:** 10.3390/pathogens10111493

**Published:** 2021-11-16

**Authors:** Germán J. Medina-Rincón, Sebastián Gallo-Bernal, Paula A. Jiménez, Lissa Cruz-Saavedra, Juan David Ramírez, María Juliana Rodríguez, Ramón Medina-Mur, Gustavo Díaz-Nassif, María Daniela Valderrama-Achury, Héctor M. Medina

**Affiliations:** 1 School of Medicine and Health Sciences, Universidad del Rosario, Bogotá 111221, Colombia; SGALLOBERNAL@mgh.harvard.edu (S.G.-B.); mariad.valderrama@urosario.edu.co (M.D.V.-A.); hmedina@lacardio.org (H.M.M.); 2 Department of Radiology, Massachusetts General Hospital, Boston, MA 02114, USA; 3 Department of Radiology, Harvard Medical School, Boston, MA 02114, USA; 4 Centro de Investigaciones en Microbiología y Biotecnología-UR (CIMBIUR), Facultad de Ciencias Naturales, Universidad del Rosario, Bogotá 111221, Colombia; paulaandr.jimenez@urosario.edu.co (P.A.J.); lissa.cruz@urosario.edu.co (L.C.-S.); juand.ramirez@urosario.edu.co (J.D.R.); 5 Division of Cardiology, Fundación Cardioinfantil-Instituto de Cardiología, Bogotá 110131, Colombia; mjrodriguez@cardioinfantil.org (M.J.R.); ramon8809@hotmail.com (R.M.-M.); 6 Division of Gastroenterology and Liver Diseases, Fundación Cardioinfantil-Instituto de Cardiología, Bogotá 111221, Colombia; drdiaznassif1@hotmail.com

**Keywords:** Chagas disease, *Trypanosoma cruzi*, vector-borne disease, heart failure, cardiomyopathy, achalasia, megacolon

## Abstract

Chronic manifestations of Chagas disease present as disabling and life-threatening conditions affecting mainly the cardiovascular and gastrointestinal systems. Although meaningful research has outlined the different molecular mechanisms underlying *Trypanosoma cruzi’s* infection and the host-parasite interactions that follow, prompt diagnosis and treatment remain a challenge, particularly in developing countries and also in those where the disease is considered non-endemic. This review intends to present an up-to-date review of the parasite’s life cycle, genetic diversity, virulence factors, and infective mechanisms, as well as the epidemiology, clinical presentation, diagnosis, and treatment options of the main chronic complications of Chagas disease.

## 1. Introduction

Chagas disease (CD) is a parasitic infection caused by *Trypanosoma cruzi,* a protozoan mainly endemic to South and Central America. Although its exact prevalence is unknown, it is estimated that approximately 6–8 million people are infected with this parasite. While most cases resolve after the acute phase of the disease, a subgroup of patients develop a chronic phase in which many organs, including the heart, esophagus, colon, and nervous system, can be affected. This review aims to present a thorough summary of the most recent breakthroughs regarding the molecular aspects of the parasite’s interactions with human hosts and an up-to-date summary of the clinical presentation and current practices for the diagnosis and treatment of the chronic complications of CD.

## 2. *Trypanosoma cruzi*

### 2.1. Classification and Biology

*Trypanosoma cruzi* is an eukaryotic microorganism belonging to the protist kingdom. Taxonomically, it is part of the Phylum Sarcomastigophora, Sub-phylum: Mastigophora, Class: Kinetoplastea, Order: Trypanosomatida, Sub-order: Trypanosomatine, Family: Trypanosomatidae, Genus: *Trypanosoma*, Species: *Trypanosoma cruzi* [1].

Trypanosomes are part of the kinetoplastea class, a group of flagellated microorganisms characterized by the presence of an organelle with a large DNA mass called kinetoplast [2]. This unusual DNA-containing organelle is a specialized region of a single mitochondrion located near the basal body of the flagellum, which contains a high concentration of extranuclear DNA, including approximately 16–30% of the mitochondrial genome. It has been suggested that its primary function is to encode respiratory chain proteins and guide RNAs for gene editing [3]. It is in close spatial relationship with the parasite’s flagellum, which has a role in intracellular infection [4].

*T. cruzi* is a highly adaptable microorganism, which undergoes several metabolic and structural variations in response to environmental stressors during its reproductive cycle. These variations occur in the different stages of its life cycle and include changes in its morphology, location of the kinetoplast, expression of surface proteins, mechanism of evasion, and reproductive capacity [3,5].

**Figure 1:**
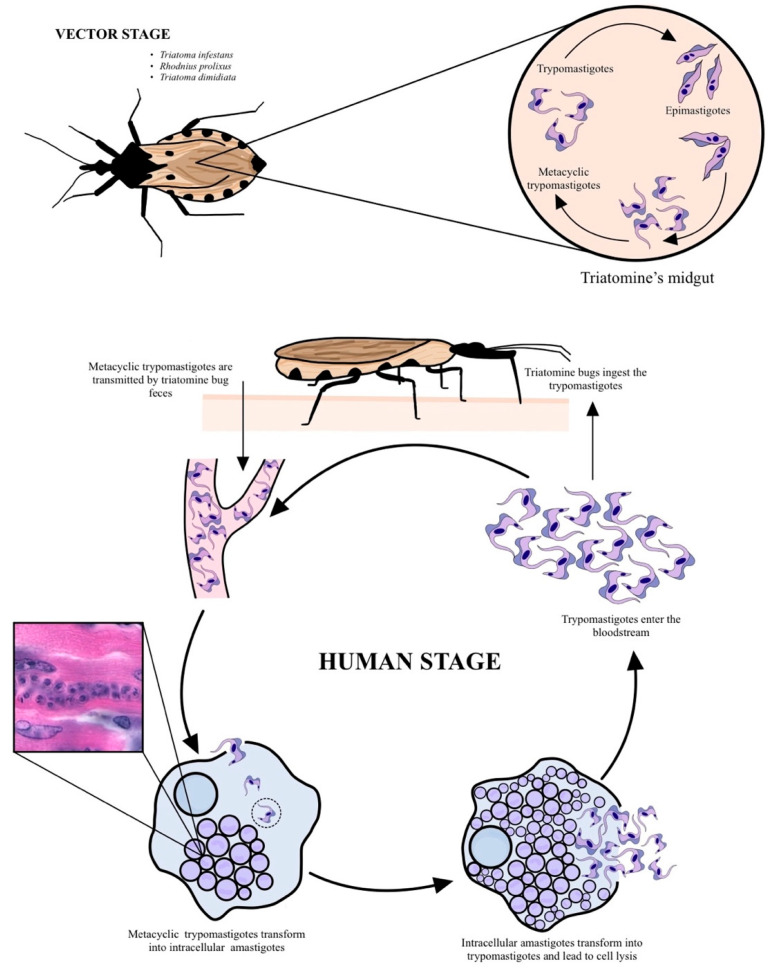
*Trypanosoma cruzi* life cycle. *T. cruzi’s* life cycle consists of four stages: epimastigotes, amastigotes, metacyclic trypomastigotes, and cell-derived trypomastigotes. Its life cycle takes place in both vectors (e.g., *Triatoma* spp.), and human hosts.

*T. cruzi* has a complex life cycle that occurs between mammalian hosts and triatomine vectors. Four well-differentiated stages have been described: epimastigotes, metacyclic trypomastigotes (both taking place in the Reduviid insect), amastigotes, and cell-derived trypomastigotes found in the host (Figure 1). The cycle begins when the vector ingests blood infected with cell-derived trypomastigotes. Once inside the vector, the trypomastigotes differentiate into epimastigotes that move across the intestine until they reach the rectal ampulla, where they transform into metacyclic trypomastigotes (infective stage). The cycle continues when the vector feeds from a mammal host. It then excretes the metacyclic trypomastigote-containing feces that pass across the bite site, infect blood mononuclear cells, and differentiate into amastigotes. After several rounds of replication, they transform into cell-derived trypomastigotes that invade new cells [6].

*T. cruzi* constitutes an heterogeneous specie and genetic variability has been observed within specimens. Currently, they are classified based on discrete typing units (DTUs), which group strains with identical genetic properties. They are identified with different immunological, biochemical, and molecular markers. Several DTUs have been described, including Tc-I to Tc-VI, and TcBat, a new recently-described genotype found to be infectious to humans through its identification in a 5-year old female living in a sylvan region in Colombia [7,8]. The different DTUs have been related to specific epidemiological cycles, transmission mechanisms, geographical distributions, vector dispersal, and CD clinical manifestations. However, further studies have shown that these associations are not always conclusive [7,9].

### 2.2. T. cruzi’s Genetic Diversity

A wide biochemical and genetic diversity has been described. Even inside a specific geographic region, *T. cruzi* has variations in its molecular and pathophysiological properties such as tissue tropism, replication rates, and infectivity. It has also been suggested that its genetic plasticity could be related to the modulation of virulence among different strains, impacting infectivity and clinical severity [10,11].

Despite intense discussion, *T. cruzi* is currently considered a clonal parasite. However, studies using different genetic approaches have suggested that *T. cruzi* has some degree of natural recombination [5,12]. Recently, genomic studies have found evidence of meiotic sex mechanisms in the genome of Ecuadorian and Peruvian *T. cruzi* clones [13]. Nevertheless, in vitro evidence suggesting sexual genetic recombination in this parasite is scarce and non-conclusive. For example, the presence of fused-hybrids after Vero cells infection and overexpression of RAD51 (protein related with meiosis and recombination) in the epimastigotes of hybrid strain CL-Brener (TcVI) has been described [14,15]. Some authors suggest that recombination might occur between epimastigotes in the digestive tract of the triatomines, considering the favorable conditions for genetic exchange secondary to environmental stress. Moreover, the meiosis-like recombination observed in *Leishmania,* and the complete meiosis in *Trypanosoma brucei,* which occurs in the salivary glands of Tse-tse flies, further supports the possibility of genetic recombination in T. cruzi [16,17].

While generally described as a diploid eukaryote, *T. cruzi’s* genetic information is organized in homologous chromosomes that change in number and size between the different strains [13,18]. The number of chromosomes changes depending on the strain and DTUs; 43 to 48 chromosomes have been described so far. The length of each chromosome is strain-specific and depends on different processes that involve insertions, deletions, and duplications of individual genes or complete genome segments [18,19].

*T. cruzi* has a complex genomic architecture, described as “genome compartmentalization”. The core compartment contains the most conserved genes. The disrupted compartment represents around 50% of all the genetic information and corresponds to repetitive sequences, represented by multigene families, such as, transialidases, mucins, mucin associated proteins (MASP), dispersed gene family proteins (DGF), and retrotransposon hot spot (RHS) [20]. These components play an essential role during *T. cruzi’s* life cycle [21] and the transcriptional and post-transcriptional processes modulating the chromatin structure as can be seen in *T. brucei* [22]. 

Additionally, *T. cruzi* has an uncommon gene expression system in which gene transcription is based on polycistronic transcriptional units, where a set of genes that are not necessarily related are transcribed at the same time [23]. Transcriptomic studies have shown differences between the gene expression across the parasite’s life cycle [24,25]. Each stage has a specific transcriptomic profile related to replication, invasion, and immune response escape demonstrating a high degree of adaptability in response to environmental stressors [21,26]. Moreover, different DTUs and virulent and non-virulent strains have shown specific repertoires of expressed genes that influence strain-specific biological properties and clinical behavior [27,28]. 

### 2.3. Virulence Factors in T. cruzi 

Several virulence mechanisms have been described in *T. cruzi’s* infectious process, and the following section aims to provide a short description of them. For this purpose, the main virulence factors will be described chronologically and following the usual course of interaction with the host: resistance to oxidative damage and evasion of the host immune response; and adhesion molecules, cell invasion and phagolysosomal escape.

### 2.4. Resistance to Oxidative Damage and Evasion of Host Immune Response

**Figure 2:**
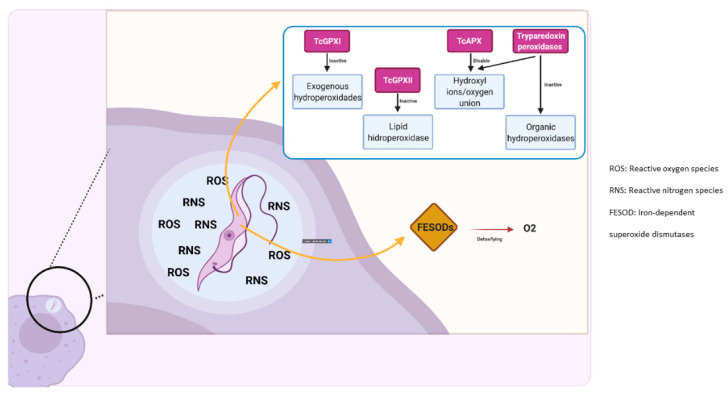
Resistance to oxidative species. *T. cruzi* has several enzymes destined to deactivate reactive oxygen species, namely, peroxidases, iron superoxide dismutases, and hydroperoxidases. These enzymes allow for the parasite’s survival and decrease the efficacy of the host’s immune response.

*T. cruzi* has adapted several antioxidant mechanisms to inactivate reactive oxygen and nitrogen species released by the host at the early stage of the infection [29]. Using a variety of enzymes such as peroxidases, the metacyclic trypomastigotes are able to evade oxidative stress caused by phagocytic cells, such as macrophages and dendritic cells [30]. Glutathione peroxidase TcGPXI, which is located in the cytosol, inactivates exogenous hydroperoxidades. Peroxidase TcGPXII present in the endoplasmic reticulum (ER), inactivates lipid-hidroperoxidase, while TcAPX disables the binding of hydroxyl ions with oxygen in conjunction with tryparedoxin peroxidases (TcCPX, TcMPX situated in cytosol and mitochondria respectively) [31]. Additionally, *T. cruzi* has four types of iron superoxide dismutases (FESOD) located in the cytosol, mitochondria and glycosomes, responsible for detoxifying reactive oxygen species [32] (Figure 2). It has also been recognized that the expression of these enzymes is strongly correlated with the parasite’s life cycle [33]. 

The trypomastigote decay accelerating factor (T-DAF) regulates the expression of C3 convertase found in all three pathways of complement: classical, alternative, and lecithin-activated. *T. cruzi* complement regulatory protein (CRP), also known as gp160, is unique to trypomastigotes and has the ability to block complement pathways and escape opsonization. CRIT (C2 receptor inhibitor trispanning protein) blocks the lecithin pathway and prevents the activation of complement. Also, TcCRT (Calreticulin), a surface molecule that inhibits the classical pathway, captures C1 and promotes parasite infectivity [33,34,35]. Of note, *T. cruzi* has proline racemases (PR) enzymes, TcPRACA and TcPRACB (*T. cruzi* proline racemase A and B, respectively), essential for eluding an immune response. Molecules such as Tc52 which suppress T cell proliferation and activation, have been described [32].

### 2.5. Adhesion Molecules, Cell Invasion and Phagolysosomal Escape

Cell invasion by *T. cruzi* is essential for its survival inside the host. This protozoan has developed surface proteins—such as transialidases, mucins, mucin-associated surface glycoproteins, and phospholipases—which allow the adhesion of metacyclic trypomastigotes and extracellular amastigotes to host cells through carbohydrate interactions [36]. 

A wide variety of genes encoding surface proteins have been described in *T. cruzi*, which in turn are recognized as virulence factors [21,37]. Recent molecular studies have shown that coding families of these genes simultaneously express protein variants, a phenomenon thought to confer the parasite an ability to evade the host’s immune response [18,38]. 

Transialidase enzymes (TS) are virulence factors whose diversification and large number of coding genes are well known. TS’s function is the acquisition of sialic acid from host cells to modify trypomastigote’s surface proteins, rendering them capable of inducing cell paralysis and cell lysis. As they are excreted in abundant quantities, it is difficult for the host to mount a neutralizing humoral response. Therefore, it is thought that the ability to excrete transialidases in high quantities is associated with increased virulence of strains [39,40,41]. Additionally, within *T. cruzi’s* genome, large gene family diversification has been found. This genomic diversity evolved as a response to the intense immunological pressure exerted by the parasite’s hosts [18]. 

**Figure 3:**
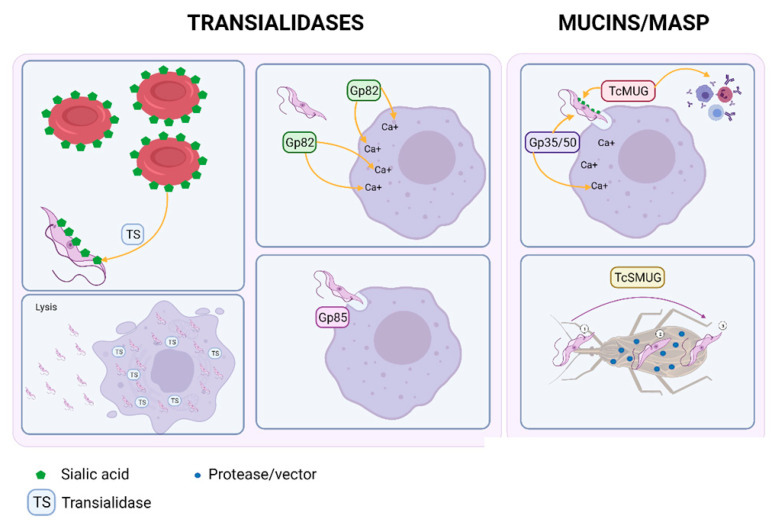
Adhesion molecules, cell invasion, and phagolysosomal escape. T. cruzi is able to invade host cells and phagolysosomal mechanics through specialized proteins such as mucins, mucin-associated proteins, transialidases, and phospholipases; allowing for carbohydrate and peptidic interactions increasing infectivity and dwindling immune response.

Surface glycoproteins expressed by metacyclic trypomastigotes such as Gp82 (transialidases-like protein) aid host cell invasion by activating intracellular Ca^2+^ signaling cascades [32,42]. This has also been described as a critical step in the union to gastric mucin in the oral route of infection [33,43]. Notably, it has a conserved FLY domain, which helps the parasite activate host extracellular signals that facilitate infection with a tropism for endothelial cells, such as heart vessels [32,33,44] (Figure 3). 

Mucins, another surface protein family is in charge of providing protection from the vector’s and human host’s immune mechanisms and enhance the invasion of specialized tissue cells [45]. They are classified in two groups: one of them is present in only mammalian hosts known as TcMUC, protecting *T. cruzi* against the host immune system and helping the adhesion by using sialic acid from the transialidases. The second one, known as TcSMUG, protects *T. cruzi* against digestive proteases from the vector [46] (Figure 3).

MASPs (Mucin–associated surface proteins) found in metacyclic trypomastigotes and bloodstream trypomastigotes promote *T. cruzi’s* invasion, replication, and survival [47,48]. Gp35/50, a protein complex that belongs to the mucins family, is essential for the internalization of the parasite through calcium-mediated pathways (Figure 3). Similarly, surface antigen TSSA (Trypomastigote small surface antigen) is a *T. cruzi* antigenic polymorphic mucin-like molecule involved in the parasite’s adhesion to host cell surfaces. Recently, TcMUC, O-glycosylated Thr/Ser/Pro-rich mucin molecules located on the *T. cruzi* surface have been associated with different host-pathogen interactions. They act as adhesion molecules that vary across the different DTUs. In addition, two different forms of TSSA have been reported, including TSSA-CL (mainly with adhesive properties) and TSSA-Sy [37,38,49]. 

Additionally, cysteine endopeptidases are essential in the infection process [50]. When *T. cruzi* invades host cells, it escape from the phagolysosome of phagocytic cells. To rupture the membrane of the phagolysosome *T. cruzi* uses a protein known as Tc-Tox, which forms a pore in the vacuole membrane, releasing the parasite and facilitating its invasion and replication [32].

The plasticity of *T. cruzi’s* genome to generate multiple variants of these proteins through gene duplication, mutation, and recombination confers great antigenic diversity. In addition, having a genetic structure rich in repetitions and retrotransposons facilitates these processes by dispersing these genes throughout the genome [18,51]. These factors increase its fitness and survival, promoting evation of the host immune response by creating several antigens. Thus, mounting a solid immune response against this huge repertoire of antigens is not easy and usually results in immune evasion by the parasite. 

### 2.6. T. cruzi Infection Models to Understand Tissue Tropism

Since *T. cruzi* strains were described, several studies have been aimed to describe and explain the parasite’s tissue tropism. Tissue distribution of protozoa has been characterized in different models to understand the pathogenesis of the inflammatory processes and the host-parasite interactions. *T. cruzi* murine models have shown distinct aspects of CD. Chronically infected mice reveal preferential patterns of tissue distribution. Studies by Andrade et al. showed a differential parasitic tissue load between the different strains used in murine infection models (Col1.7G2 and JG, which belong to *T. cruzi* I and *T. cruzi* II lineages). Parasites were detected in cardiac tissue when mice were infected with either lineage of *T. cruzi*. Notwithstanding, when mice were infected with both strains, only the JG strain was found to invade the heart [52,53,54]. These results suggested that several factors, including the parasite’s intracellular activity, the host’s immune system (such as the Major Histocompatibility complex –MHC- locus), and the host-parasite interactions, are pivotal for determining preferential tissue colonization [55]. Myocardial tropism might be influenced by peptidic interactions conserved in transialidases such as gp85, which maintains interplay with the vascular endothelium [54].

Likewise, de Castro et al. [28] described the factors that drive the parasite to remain in some organs chronically. They used the same strains, JG and Col1.7G2, to infect mice. mRNA sequencing was performed in mouse hearts, and showed transcriptional changes in parasites, which also altered gene expression in host myocardial tissue. JG strain and mixed infections of both DTUs were associated with the downregulation of various genes related to oxidative metabolism. Col1.7G2 and mixed DTUs, showed activation of immune system-related genes denoting differences between strains in organs such as the heart [28,54]. 

On the other hand, Santi-Rocca and colleges, studied the parasite tropism in syngeneic mice infected with six parasite strains representing each DTU. q-PCR was performed to find the parasitic load in each organ. During the acute phase, the parasite load was diverse, and the protozoans were found in the spleen, gut, heart, and skeletal muscle (quadriceps); no protozoa were found in the brain or liver. In contrast, there was no evidence of parasites in the spleen, liver, esophagus, and brain during the chronic phase. Of note, VFRA strain representatives were not completely cleared during the chronic phase; as a result, all animals had a significant parasitic load in the heart during this phase. In this case, these findings highlight the association of strain determinants and the stage of infection with organ tropism and the degree of tissue damage [56].

*T. cruzi* also has a high affinity for adipose tissue, demonstrated in vivo and in vitro, by Combs et al. [57] and Ferreira et al. [58] Adipose tissue is a crucial target for parasite invasion and disease progression [57]. This protozoan can modify adipokine secretion and alter the host’s metabolic profile [54]. It has been shown in murine models that hyperglycemia increases parasitemia and mortality, and it is plausible that the parasite may cause it [58,59]. 

Using a FAT-ATTAC murine model, in which mice were inoculated intra-peritoneally with trypomastigotes to cause acute and chronic disease, Lizardo et al. [60] demonstrated the correlation of Chronic Ch-CMP progression to lose adipose tissue. During the acute phase, increased lipid accumulation in myocardial cells, infiltration of immune cells and elevated number of parasites was reported. Moreover, results on *T. cruzi* infection in chronic disease suggest that adipose ablation elevates adipogenic signaling and lipids levels in myocardial tissue in early stages. Also, cell infiltration and maintenance of the protozoan in adipose tissue modifies processes such as adipogenesis and lipolysis, which may cause adipose cell apoptosis and necrosis in infected mice. The loss of adipose cells throughout infection contributes to the progression of cardiac pathophysiology and allows an increased parasite load with further invasion of immune system cells [60]. 

## 3. Epidemiology and Genetic Variability Implications

CD is primarily endemic to tropical and subtropical countries, even though it has been reported worldwide. As for most vector-borne diseases, people in low socioeconomic strata are at greater risk of contracting the disease and suffering its chronic complications [61]. The most important risk factors linked to CD include rural or countryside residency, low educational status, and advanced age. Although it was initially believed that there was an increased disease burden in males, there is no epidemiological evidence of a gender-associated risk [62,63].

CD carries a high morbidity and mortality burden, which has a profound impact on developing countries in which healthcare access is not universal and where hindrances preventing proper diagnosis and management are present [61]. In light of these facts, CD is considered a neglected tropical disease by the World Health Organization [64]. The highly morbid chronic complications associated with the disease have been estimated to cost over USD 7.19 billions/year on healthcare-related expenses and over 806,170 disability-adjusted life-years (DALYs) [65]. The authors of the above-mentioned study attribute a significant proportion of the burden to cardiovascular disease-induced early mortality. The countries with the highest prevalence of CD are located in South America and include Bolivia, Argentina, Paraguay, and Ecuador (Table 1).

While most cases of CD are vector-borne, oral transmission has been drawing attention in recent years, mainly due to its high fatality rate secondary to an increased association with acute fulminant myocarditis and meningoencephalitis [72,73,74]. This type of infection usually presents as an acute and life-threatening condition that has been mostly tied to outbreaks of contaminated food or beverages [75,76,77]. It is thought that an overproduction of INF-gamma-inducible cytokines that promote a pro-inflammatory environment suitable for the parasite is the main reason for the oral transmitted CD to be linked with higher acute disease severity [72,77]. The majority of these outbreaks have been reported in Venezuela, Colombia, and Brazil [72,73,75,76,77,78].

In previous years, CD outside of Central or Latin America was considered a rare event and was primarily seen in travelers visiting endemic countries; nevertheless, the increase in migration seen in the last three decades has had a critical impact in the overall prevalence of CD in countries outside of Latin America, mainly in the United States of America (USA) [79]. While there is a lack of data regarding the exact prevalence of CD in the USA, various studies have suggested that from 2009 up to 2016, between 0.09 to 0.1% of the USA population was infected with CD [70,71,80]. Moreover, since blood donor screening for CD was only introduced in the USA since 2007, it is thought that many patients may have been infected and are currently asymptomatic due to blood-borne acquisition of the parasite related to blood transfusions [80]. 

On the other hand, there have been several reports of domestic infection of CD in the USA and, in many of them, *Triatoma* have been found in the surrounding areas of the patients who contracted the disease [81,82,83]. It should be noted, however, that some studies have failed to provide insights regarding the direct route through which native Triatoma species in the USA pass on CD. Particularly, *Triatoma gerstaeckeri*, *T. protracta*, and *T. sanguisuga*, which are native species of the USA, have not been linked with the classical route of infection mediated by immediate defecation after a blood meal in human hosts [84,85,86,87].

In Europe, CD is an even stranger condition [88,89]. Only Spain, France, Switzerland, Belgium, and the United Kingdom have implemented blood donor screening tests for *T. cruzi* [88,90]. In other countries, blood donor screening for CD is rare and not done routinely. A study performed in Geneva and Florence, aimed to estimate the prevalence of *T. cruzi* infection in blood donors considered high-risk in spite of their country of origin; both centers screened a large population considered at risk with 1.012 patients from Geneva and 867 from Florence. In donors migrating from Latin America, a prevalence of 12.8% and 11.3% was found, respectively [91,92]. Basile et al. [93] reported a total of 4290 documented cases of CD in Europe. In this cohort, the highest prevalence was seen in Spain with 3821 cases. Switzerland, Italy, and France followed with over 100 cases each [93]. While not systematically, other studies have also estimated the prevalence of subclinical infection in Latin American blood donors. The most remarkable ones were done in Barcelona and Milan. An estimated prevalence of 2.9% and 8.8% were found, respectively [94,95]. While CD remains a relatively infrequent disease in Europe, some authors consider the lack of systematic screening a problem for public health mainly because of globalization and the increase of solid organ transplant rates in recent years [88,96].

Though not significant enough to be considered a public health issue, it should also be noted that reports have been published regarding cases of CD described in Canada, Australia, and New Zealand [90]. Particularly in Australia, by 2011, a total of 1928 patients were diagnosed with CD, while in New Zealand as of 2006, only 98 patients had a confirmed diagnosis [97].

As mentioned above, there have been 7 major genetic lineages of *T. cruzi* discovered, being named TcI (for *Trypanosoma cruzi–I*) up to TcVI, while the last and recently discovered seventh strain named TcBat [7,98]. The most extensively studied strain is TcI which has been further subdivided into TcI_a_ up to TcI_e_; furthermore, it appears that geographic clustering is an important phenomenon with regard to genetic heterogeneity and variance amongst TcI subgroups, a fact that is thought to impact clinically regarding phenotype of chronic forms [19,99,100,101].

Additionally, it has been shown that genetic heterogeneity between lineages of *T. cruzi* plays a key role in serodiagnosis, particularly, North and Central America have less accurate tests since most of the antigens used in serologic kits are used based on South America strains. This further suggests the importance of a widespread research of genetic behavior of *T. cruzi* [102,103]. Moreover, genetic variability and low degree of genome conservation across all *T. cruzi* strains may in fact result in false negative diagnostic tests in regions where genetically diverse strain clusters are prevalent [104].

Finally, most studies investigating the effect of *T. cruzi* strains and chronic phenotypes have been done with clinical reports instead of quantitative epidemiological studies based on population data. Nevertheless, there are some clear associations that have been further studied in vivo and that are the foundation for future research of host-parasite interactions [25,105]. First and foremost, the TcI strain is mostly associated with the indeterminate and cardiac phenotype of CD, whereas gastro-intestinal (GI) involvement is rather strange. Also, it should be noted that it is mostly seen in North, Central, and the northern countries of South America [25]. On the other hand, Central and southern countries in South America exhibit a different endemicity of *T. cruzi* strains, where TcII, TcIII, TcV, and TcVI are prevalent, with TcII being the most common. In fact, countries such as Argentina, Brazil, Bolivia, Uruguay, Paraguay, and Chile have higher rates of GI megasyndromes given their higher prevalence of the latter strains of *T. cruzi* [101,106,107]. Nevertheless, it is worth mentioning that in most surveys and studies, TcII is the strain with the strongest association with GI involvement [108,109].

## 4. Chagas Cardiomyopathy (Ch-CMP)

### 4.1. Epidemiology and Pathobiology

One of the most dreadful chronic complications of CD is Ch-CMP, the most common non-ischemic cardiomyopathy in Latin America [110]. It is estimated to affect approximately 20–35% of patients with the chronic form of CD [62,111]. It is a widely variable heart disease, with multiple manifestations including heart failure, supra-ventricular and ventricular arrhythmias, heart blocks, ventricular aneurysms, ventricular thrombi, stroke, and sudden cardiac death (SCD) [62,111,112].

**Figure 4:**
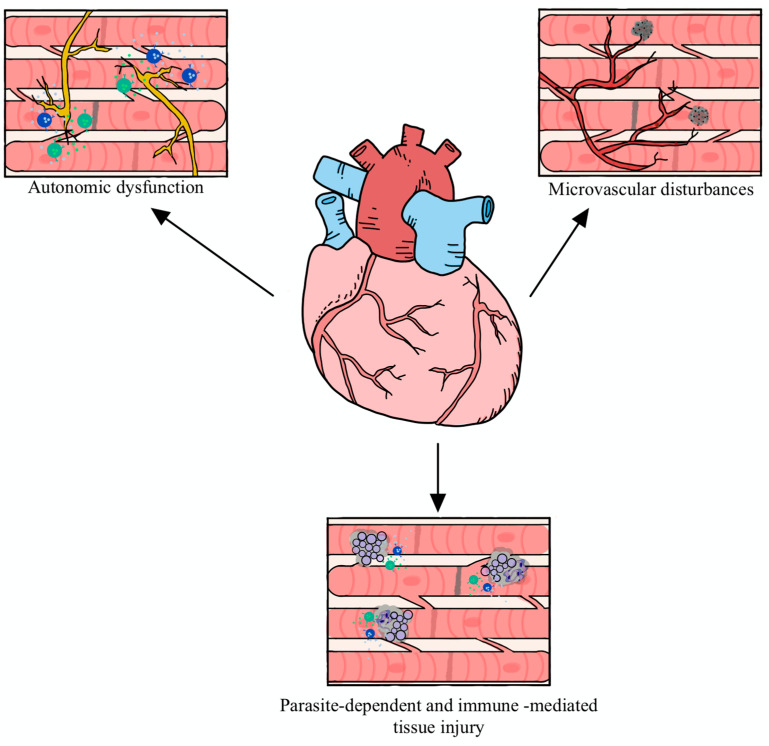
Pathophysiologic mechanisms of Chagas Cardiomyopathy. Three main mechanisms have been described: (1) autonomic dysfunction, through catecholamine excess; (2) microvascular disturbances, caused by microthrombi formation; and (3) parasite-dependent with immune mediated tissue injury, in which cytokines and inflammatory mediators exert direct tissue damage to the heart.

Several pathophysiological processes have been identified to impact the development of Ch-CMP. However, four cornerstone mechanisms play a crucial role: cardiac autonomic dysfunction, microvascular disturbances, parasite-dependent myocardial damage, and *T. cruzi* immune-mediated cardiac tissue injury (Figure 4) [113,114].

Cardiac autonomic dysfunction, the first of the pathophysiological mechanisms, impairs the parasympathetic nervous system’s ability to keep a fine-tuned control of the heart rate and rhythm [113,114]. This process is mainly caused by direct damage to intramural neurons and ganglionic damage to neuronal somas and peripheral axonal projections; moreover, parasite-induced autoimmune damage caused through CD4+ and CD8+ T-cells also contributes to the autonomic abnormalities seen in Ch-CMP [115,116]. These abnormalities are thought to cause catecholamine-induced cardiomyopathy, resulting in contractility imbalance because most of the intramural neuronal fibers are parasympathetic. Consequently, a loss of the physiologic homeometric compensation mechanisms ensues this autonomic disarray [115,116]. Additionally, the loss of parasympathetic input has been associated with the generation of malignant ventricular arrhythmias, which are the leading cause of SCD in this population [115,116,117].

Microvascular disturbances are another highly contributing factor in the complex patho-physiology of Ch-CMP. The hallmarks of this mechanism are the formation of micro-thrombi in small penetrating vessels, peri-vascular immune damage, intimal fibrosis, and myocardial “patch” micronecrosis. These factors contribute to an imbalance in blood flow caused by the disruption of the precise control of coronary blood flow, leading to micro-infarctions and, eventually, the formation of ventricular wall aneurysms [115,118]. 

An increased endothelin production and unopposed sympathetic stimulation are the main current theories supporting the microvascular disturbances seen in Ch-CMP. Moreover, the lack of diffuse epicardial histopathological evidence of necrosis further indicates that all of the abnormalities take place at the coronary microcirculation [114,119]. Given that these phenomena occur at the microcirculation, the most affected areas are distal or “watershed” perfusion areas, in particular, localized at the apex and at the basal inferolateral wall. These segments are the most commonly affected with focal fibrosis and aneurysm formation [113,119,120].

The last mechanisms responsible for the pathogenesis of Ch-CMP are a parasite-mediated myocardial injury and an immune-mediated damage elicited by the host response towards *T. cruzi.* Both of these mechanisms are deeply intertwined and share multiple molecular grounds [114]. Although many factors modify the severity of the disease and its clinical phenotype, the *T. cruzi* strain, the degree of parasitemia, the tropism for myocardial tissue, host comorbidities, and intrinsic factors of the host, including genetic susceptibility, are the main elements affecting the presentation and course of the infection [114]. 

The main entrance route of *T. cruzi* to myocardial cells appears to be mediated by saccharide residues on membrane proteins, most importantly, the family of galectins, of which Galectin-1 has been shown to modulate the entrance of *T. cruzi* to myocardial cells. [114,121]. Correspondingly, immune-mediated myocardial injury is mainly caused by a delayed (type IV) hypersensitivity reaction with a pleomorphic tissue infiltrate and myocardial cytolysis elicited by the parasite’s immunogenicity [122,123,124]. Given these facts, more emphasis is given towards the importance of parasitemia, since a higher level is associated with a greater cellular and humoral immune response [122,125]. Lastly, it is worth mentioning that in patients with Ch-CMP, autoantibodies have been found directed towards adrenergic, and muscarinic receptors, as well as to myocyte structural proteins; this data further supports the hypothesis of catecholamine-induced cardiomyopathy and the impairment of the autonomous nervous system control over the heart’s rate and contractility [126,127].

### 4.2. Clinical Manifestations, Diagnosis, and Treatment

Cardiac involvement has a significant heterogeneity regarding its onset, presentation, progression, and severity. While many factors and triggers have been described, the most important ones are age, parasite strain, innate genetic susceptibility, African ancestry, nutritional and health status, and both the degree and clearance rate of parasitemia [128]. 

Approximately 3 months after the resolution of the acute phase, some patients may exhibit electrocardiographic changes including sinus tachycardia, variable degree heart blocks, prolonged QT and/or PR interval, repolarization abnormalities, and increased incidence of premature ventricular contractions [129,130]. Moreover, the most typical cardiac electric abnormalities are right-bundle branch block (RBBB) accompanied by a left anterior fascicular block, both of which are highly specific yet not sensitive findings [129,130]. Clinically, patients present with a highly variable array of symptoms including, but not limited to, dyspnea, orthopnea, fatigue, dizziness, chest pain, palpitations, syncope, nausea, vomiting, diaphoresis, and SCD [131,132]. Upon examination, non-specific findings are usually detected, including extra heart sounds (S3 and S4), mitral murmurs due to functional regurgitation, and wide splitting of S2 secondary to RBBB [132,133,134]. 

Chagas disease manifestations can be broadly divided into three main categories: cardiac rhythm abnormalities, ventricular dysfunction, and thrombo-embolic events. The first of them is characterized by rate and rhythm abnormalities including both bradyarrhythmia and tachyarrhythmia, being ventricular arrhythmias the most common and life threatening [135]. It is estimated that 50 to 65% of patients with cardiac involvement will develop ventricular arrhythmias [136,137]. These patients frequently present with either monomorphic or polymorphic ventricular tachycardia in the setting of chest pain, dizziness, confusion, palpitations, or dyspnea [138,139]. In some of these patients, autonomic symptoms may be absent due to the destruction of sympathetic and parasympathetic nerve endings. Finally, SCD is one of the leading causes of death in patients with a cardiac phenotype of chronic CD, accounting for almost 60% of all deaths [140,141,142]. 

Regarding ventricular dysfunction, most of the patients eventually develop low left ventricular systolic function quantified through left ventricular ejection fraction (LVEF), usually preceded by segmental wall motion abnormalities in the left ventricle; additionally, a smaller subset of patients may develop biventricular dysfunction with left ventricular remodeling preceding right ventricular involvement [143,144]. Symptoms due to right and left ventricle failure include, fatigue, dizziness, chest pain, dyspnea, palpitations, cough, peripheral edema, ascites, and hepatomegaly [62]. Ch-CMP is currently classified from stages A to D upon the severity of symptoms and the evaluation of structural abnormalities through imaging studies (Table 2) [145].

Lastly, thrombo-embolic events are a frequent cause of disability and/or death in patients with Ch-CMP. In CD-endemic areas, Ch-CMP-related strokes constitute 18% of all cases [146]. Additionally, pulmonary emboli, although uncommon, have also been documented. Thromboembolic events are thought to be caused by a sum of both ventricular thrombi—caused due to wall motion abnormalities and aneurysm formation—and atrial thrombi—caused by chamber enlargement and non-laminar flow [147,148,149]. While most of Ch-CMP stroke patients present with anterior circulation syndromes with cortical motor and sensory deficits, up to 30% of patients may present with posterior circulation syndromes, affecting the occipital lobe, cerebellum, and brainstem [150,151].

The diagnosis of Ch-CMP in patients with confirmed CD is done through a multimodal approach including clinical examination and imaging stratification. In patients with a clear history of CD, a positive serological test and an unequivocal clinical presentation, the diagnosis of Ch-CMP can be presumed, and medical care established, aiming at identifying possible complications, diminishing disease progression, and treating the most clinically significant symptoms [145]. This is mainly done using ECG, a trans-thoracic echocardiogram (TTE), and a 24-h Holter analysis if any abnormality is seen in the standard ECG, or if there is any suggestion of atrial fibrillation or ventricular tachycardia [145,152].

**Figure 5:**
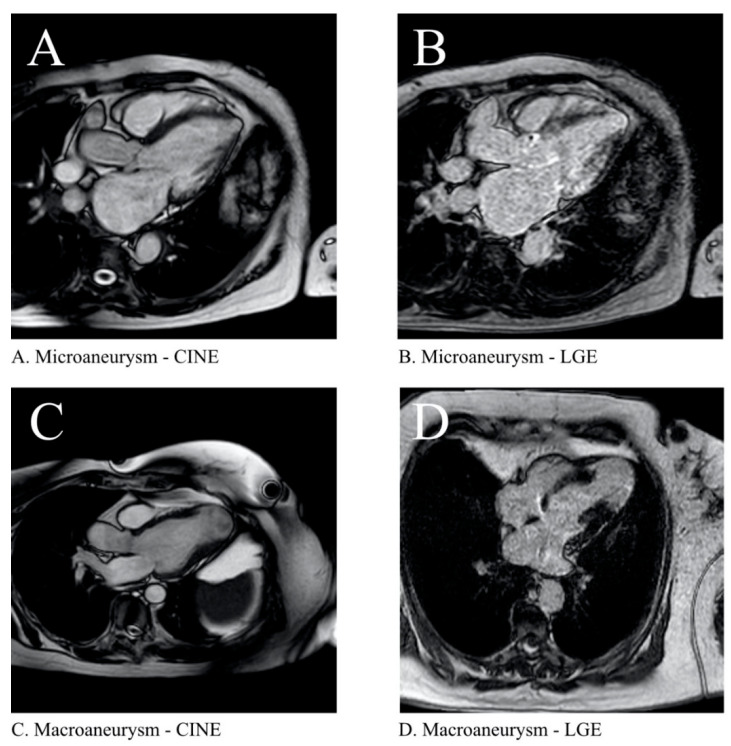
Cardiac Magnetic Resonance Imaging in Chagas disease. (CINE: Steady state free precession sequence. LGE: Late gadolinium enhancement).

Finally, cardiac magnetic resonance imaging (CMR) has recently shown promising results in the study of Ch-CMP [153]. This imaging modality has proven to assess with high precision and accuracy the systolic function of the both ventricles in patients with Ch-CMP with equal or even superior results compared to TTE [154,155]. Moreover, recent studies conducted in tertiary care centers have shown that late gadolinium enhancement analysis (LGE) can be used for optimal assessment of disease severity and can also act as a surrogate for prognosis in patients with overt Ch-CMP depending on the amount of scar found [113,156,157,158,159,160]. For over 20 years, tissular edema identified both by traditional black blood imaging sequencing and more recently using T1 and T2 mapping, has also been described in Ch-CMP although its clinical application is still to be determined [161,162,163,164]. Nonetheless, an important limitation for CMR wide use of Ch-CMP evaluation is its availability in Latin America and the high costs it entails for healthcare systems when compared with TTE. In Figure 5, panel A and B represent the cine and LGE images of the same patient with an apical ventricular micro-aneurysm with scar in the same location. Of note, there is also transmural scar in the basal inferolateral wall. Panel C and D represent a larger apical aneurysm in a different patient in cine images with corresponding scar during LGE. 

As with any other chronic cardiac condition, treatment of Ch-CMP is mainly done through an interdisciplinary approach in which lifestyle modifications summed with pharmacological treatment constitute the foundations of management. A remark is due respecting the use of anti-trypanosomal agents in Ch-CMP. Currently, there is a lack of evidence supporting the use of these medications as part of the integral treatment of cardiac involvement in CD. One of the most relevant trials assessing this clinical question was the BENEFIT trial. In this multicenter, international, controlled clinical trial, 2854 patients with Ch-CMP were randomized to receive either benznidazole or placebo between 2004 and 2011. While a clear reduction in parasite load was seen in the benznidazole group, there were no changes in the clinical outcomes, including mortality, SCD, requirement of pacemaker or implantable defibrillator insertion, heart transplant, onset of heart failure, and stroke. In light of these findings, excluding very few exceptions, anti-trypanosomal agents are not recommended as part of the mainstay treatment for Ch-CMP [165].

Currently, the main pharmacological agents used in Ch-CMP are derived from evidence extrapolated from studies done in broader population samples with congestive heart failure and no CD. Mainstay therapy for these patients is based on neuro-hormonal blockade and SCD prevention [166]. As such, patients should be initiated on an angiotensin-converting enzyme inhibitor (ACE-I) or angiotensin II receptor blocker (ARB), a β-blocker, and a mineralocorticoid receptor antagonist (MRA) for patients with NYHA III or IV functional class, or on the basis of LVEF assessment [166]. Although evidence is scant regarding the use of dual angiotensin receptor and neprilysin inhibitors (ARNI) and sodium-glucose transporter 2 inhibitors (SGLT-2), clinical trials are currently ongoing given the fact that approximately 7.6% of patients enrolled in the PARADIGM-HF, and the ATMOSPHERE trials, were diagnosed with Ch-CMP. Both medications are promising options for the treatment of Ch-CMP, an otherwise often refractory and highly morbid cause of heart failure [167,168,169,170]. Likewise, SCD prevention is based in the insertion of an implantable cardiac defibrillator and amiodarone therapy [171,172,173]. This strategy has proven to be effective and safe, reducing all-cause mortality in up to 72%, and the incidence of SCD in up to 95% when compared with amiodarone therapy alone [172]. 

Finally, given the high morbidity and mortality of patients with advanced heart failure secondary to CD, advanced interventions should be considered in patients with refractory symptoms. Although very few studies have been conducted, some experimental evidence suggests that the use of mitral clip in Ch-CMP as well as left ventricular assistance devices might prove valuable for the management of end-stage heart failure due to CD [174,175,176]. On the other hand, heart transplantation remains another option for the treatment of advanced Ch-CMP [177,178,179]. Nevertheless, the risk of infection reactivation in the context of immunosuppression lingers and evidence regarding its prevention, diagnosis, and treatment is scarce [180,181,182,183].

## 5. Gastrointestinal and Visceral Involvement

### 5.1. Epidemiology and Host-Parasite Interaction

Gastrointestinal (GI) involvement is typical of CD. Although, the esophagus and colon are the most commonly involved segments, virtually any portion can be affected [184]. Approximately 30% of patients with the chronic indeterminate form of CD will eventually develop cardiac involvement, GI involvement, or both [185]. However, GI involvement remains a less frequent complication in patients suffering from reactivation after a solid organ transplant or in immuno-suppressed patients, with around 10 to 21% of patients developing GI complications [186,187]. 

The incidence of GI involvement in CD is highly dependent on the geographic location. It is thought that the distribution of different genotypes of *T. cruzi* accounts for its variability in patients with chronic CD in endemic regions [188]. Also, opposite to Ch-CMP, GI involvement is not associated with higher mortality rates although it entails a profound impact upon the quality of life.

The hallmark finding of *T. cruzi’s* damage to the GI tract is the denervation of both the myenteric and sub-mucosal plexus through a parasite and immune-mediated neuronal destruction [189,190]. Other findings include focal areas of fibrosis and an inflammatory infiltrate consisting of lymphocytes and eosinophils mainly. Additionally, as a compensatory mechanism, tissue samples of affected areas are remarkable for hypertrophy of the muscularis mucosa [184,190]. 

While not completely understood, the pathophysiology of GI involvement in chronic CD encompasses a wide array of factors both from the host and the parasite. A current hypothesis supports the fact that denervation of the GI tract is mostly due to an abnormally exaggerated immune response elicited against the parasite. It has been shown that an inverse correlation exists between the number of cytotoxic cells (natural killer cells, CD8+ T-cells, and macrophages) and the number of viable neurons in patients with chagasic megaesophagus [191]. Also, a direct relationship has been described regarding the presence of megaesophagus and the presence of parasitic kDNA, with 100% of samples from patients with megaesophagus being positive for kDNA, while only 60% of patients with chronic CD but without overtly clinical GI involvement had a positive result for kDNA [191]. Similarly, a higher parasite load has been associated with a higher progression rate of the GI involvement, as well as with a higher degree of inflammation evidenced by an increase in inflammatory infiltrates of affected tissues and higher concentrations of inflammatory cytokines including TNF-alpha, INF-gamma, IL-2, IL-17, and IL-6 [192]. The main theory behind the inflammatory-eliciting activity of the parasite, lies behind a cross-reactivity between parasite antigens, and neuronal surface proteins from the enteric nervous system, a theory supported by the fact that certain strains of *T. cruzi* are linked with a higher strength to the development of gastrointestinal disease [192,193]. Additionally, recent research has shown that the NOD2 receptor may be protective against GI involvement in CD, given the fact that in murine animal models, NOD2 deficient specimens exhibited reduced intestinal motility, and chronic increase in bowel thickness as well as higher inflammatory mediator concentrations in sampled tissues when compared with wildtype animals without NOD2 receptor mutations [194]. 

Another interesting factor that has drawn attention of researchers in the past decade is the gut dysbiosis caused by CD. It has been hypothesized that a relationship between the patient’s microbiome and the phenotype of the chronic form of CD exists [195,196]. For instance, De Souza-Basqueira et al. [196] performed a stool microbiome analysis in 104 chronic CD patients with differing phenotypes (including cardiac, GI, and indeterminate) and compared them with that of 31 controls. While no statistically significant differences were found amongst the CD groups and the controls, a remarkably lower representation of Verrucomicrobia, particularly, of the Akkermansia genus, was found in the Chagas groups and even more so, in the cardiac phenotype group [196].

Further evidence from a study conducted in Bolivia through oral, skin, and stool microbiome analysis both, before and after treatment with benznidazole of 20 infected children with CD, resulted in similar results [195]. In this study, the cases were selected from a 543-patient screening sample of which 3.7% (the 20 cases) were positive. The comparison was made with 35 control children, which were seronegative for CD. Results showed that patients infected with *T. cruzi*, had fecal Firmicutes and lower Bacteroidetes compared to controls. Furthermore, microbiome analysis would become similar after infected patients were treated with benznidazole. Nonetheless, oral and skin microbiomes remain altered after the treatment with anti-trypanosomal therapy. These findings support the thesis that an altered microbiome could play a role in the pathogenesis and phenotype of the chronic forms of CD [195].

Finally, although it escapes the scope of the current review, a remark is due regarding recent research upon vector-parasite-microbiota interactions [197]. This tripartite approach points towards a better understanding of all the molecular aspects that play a role in the pathogenicity and clinically relevant phenotype variation that has been thoroughly reported thus far. One of these compelling findings was recently published by Díaz et al. [198] showing that the microbiome of six different species of *T. cruzi* vectors (*P. megistus, R. prolixus, T. brasiliensis, T. infestans, T. juazeirensis, and T. sherlocki*), was dramatically different and exhibited a species-specific variation when infected with *T. cruzi* strain 0354 epimastigotes [198].

### 5.2. Clinical Manifestations, Diagnosis, and Treatment

Gastrointestinal manifestations in CD are primarily seen in the chronic phase of the disease. Nevertheless, patients during the acute phase have been shown to present temporary and nonspecific symptoms such as dysphagia, diarrhea, abdominal pain, and a retrosternal burning sensation [199]. In the chronic form, patients develop symptoms between the third and fifth decades of life, and as previously mentioned, the esophagus and colon (remarkably, the rectum, sigmoid colon, and descending colon) are the most commonly affected portions [184,200]. 

### 5.3. Salivary Glands

An ubiquitous symptom found in patients with gastrointestinal involvement is sialorrhea [201]. As with other esophageal disorders like idiopathic achalasia and eating disorders such as bulimia and anorexia nervosa, the parotid gland undergoes hypertrophy, probably as a compensatory mechanism for the dysfunctional motility [202,203]. 

### 5.4. Esophagus

More than half of patients that course with CD in the indeterminate phase and that are apparently asymptomatic have impaired esophageal motility. When not actively suspected, esophageal involvement in CD is often confused with idiopathic achalasia, given the clinical similarities between both conditions. The most often described symptoms include dysphagia, odynophagia, epigastric pain, and regurgitation [200,204]. In severe cases of megaesophagus, malnutrition and weight loss are frequently present. A critical distinction between idiopathic achalasia and chagasic megaesophagus is the resting pressure of the lower esophageal sphincter, measured through high resolution esophageal manometry. In achalasia, the loss of inhibitory innervation creates a higher resting pressure compared to that of healthy subjects. In chagasic megaesophagus, however, both excitatory and inhibitory stimuli are lost; thus, the resting pressure of the lower esophageal sphincter is even lower than that of healthy subjects. This pathophysiological component will provide insight into how treatment effectiveness, although similar in nature and approach, may differ in both conditions [205,206].

The diagnosis of chagasic megaesophagus is usually made in a multi-step manner, with barium esophagogram, upper endoscopy, and esophageal manometry being the mostly used diagnostic tools. Usual findings upon barium esophagogram include dilation of the esophagus, “bird-beak” or “hummingbird” appearance of the lower esophageal sphincter, and delayed emptying of barium contrast [207]. Additionally, four stages of severity have been proposed for the evaluation of megaesophagus (Table 3).

As mentioned above, the use of upper endoscopy has also been proposed as a tool for evaluating chagasic megaesophagus and a staging system has also been established. However, given the fact that it has a low sensitivity and high specificity, other techniques are preferred such as esophagography are preferred [208].

Lastly, the treatment of chagasic megaesophagus is based on decreasing the lower esophageal sphincter pressure mainly through direct inhibition of smooth muscle contraction. This can be achieved with pharmacological agents or surgery. The most commonly used medications are isosorbide and nifedipine, both of which have shown to be effective in decreasing symptoms. Although isosorbide has a higher effectiveness, its side effect profile decreases patients’ adherence, making nifedipine the usual first-line alternative for these patients [209]. Stage I, II, and III megaesophagus can be directly managed with pneumatic dilation or surgery. Stage IV, however, carries with itself a high risk of rupture and thus contraindicates mechanical dilation procedures; in this subset of patients, a Heller myotomy or an esophagectomy might be the only interventions able to relieve symptoms [184,210,211].

### 5.5. Stomach

Though “megastomach” has been described, it represents a rare and for the most part, clinically insignificant condition. Moreover, gastric motile and secretory abnormalities have also been reported [212]. For instance, gastric acid secretion upon stimulation and at rest has been shown to be decreased compared with healthy subjects [213,214]. Regarding motility, gastric dysrhythmias seen through electrogastrography studies have been proven to present more often in patients with CD compared to controls [215]. Moreover, gastric emptying is delayed after solid meals, while liquid meals appear to have a faster emptying [212,216]. 

The pathophysiology behind these findings is thought to be the damage of excitatory neurons in the stomach and the impairment of inhibitory neuronal impulses. The excitatory tone is responsible for food transit following a solid meal, in conjunction with finely tuned secretor functions of the stomach’s parietal and chief cells. Consequently, the impairment of excitatory impulses is, for the most part, responsible for the delayed emptying after solid meal ingestion [217]. Inhibitory tone accounts for gastric accommodation after the entrance of meal contents, and thus, given the impairment of inhibitory impulses, the stomach is unable to relax, and liquid meals are rapidly emptied [212,216].

### 5.6. Gallbladder and Biliary Tract

A fascinating finding has been demonstrated upon histopathological examination of the gallbladder of patients with gastrointestinal involvement in chronic CD. A reduced neuronal count on the gallbladder wall has been shown when compared with healthy controls [218,219]. Additionally, the prevalence of cholelithiasis is increased in patients with CD, given the motility impairment of the gallbladder [220,221]. Moreover, the lack of inhibitory neurons in the gallbladder wall has been associated with an increased sensitivity to cholecystokinetic agents through scintigraphy gallbladder emptying studies in which earlier, more intense, and longer-lasting gallbladder contractions [219]. 

### 5.7. Small Bowel

While exceedingly uncommon, megasyndromes affecting the small bowel have also been described in patients with CD and are associated with a profound impact in the quality of life [216,222]. Particularly, megaduodenum and megajejunum have been described in some case series and reports [222,223]. The most noticeable physiologic abnormality has been shown to be motility delaying, a finding that was evidenced through manometric studies, which revealed a substantial slowing of the propagation of motor impulses [224].

The most frequent complications are bacterial overgrowth, steatorrhea, chronic diarrhea, and malabsorptive syndromes [222,223]. Moreover, an increase in carbohydrate absorption has also been described as chagasic enteropathy [225,226].

### 5.8. Colon and Rectum

The colon, more specifically, the descending and sigmoid colon, and the rectum, are the second most common segments of the gastrointestinal tract, after the esophagus, affected by CD. The most common presentation of colonic involvement of CD is chronic constipation. However, other symptoms such as diarrhea, bloating, colicky abdominal pain, and rectal tenesmus are also frequent [227]. In terms of pathophysiology, colonic neuronal plexuses (submucosal and myenteric) are destroyed, thus, decreasing both excitatory and inhibitory impulses in the affected segments [189,228,229,230]. This process decreases basal motility in the sigmoid and rectum areas as well as decreased wave frequency propagation evaluated using manometry [189,231]. This denervation has also been associated with a lack of relaxation of the internal anal sphincter, both findings contribute to chronic constipation seen in the most patients with colonic involvement [227,232].

An interesting finding in patients with colonic involvement is the decreased risk of developing diverticular disease. While patients with megacolon did have diverticula, the vast majority of them were located in non-affected segments, further suggesting that dilated areas of the colon are inimical to the development of diverticula [233].

**Figure 6:**
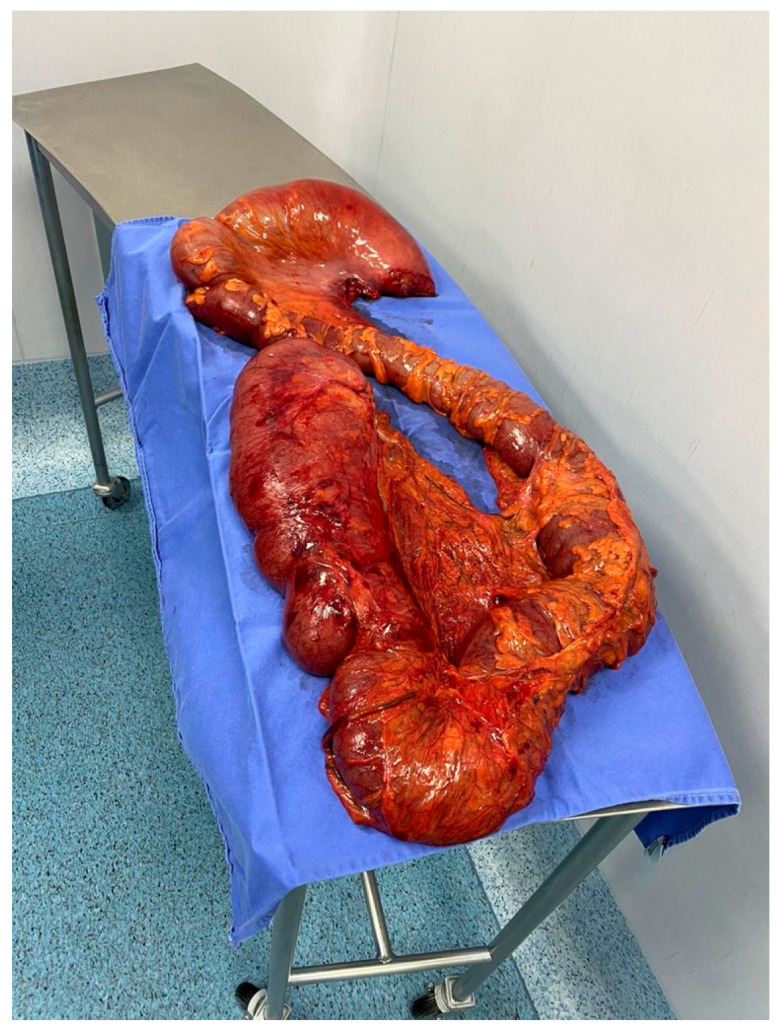
Gross Pathology: Chagasic Megacolon.

Diagnosis of chagasic megacolon is mainly clinical. However, when a confirmatory test is needed, plain abdominal radiography may show dilated intestinal shadows with increased luminal air. In contrast, enhanced imaging such as contrast-enhanced computed tomography or barium enema, decreased bowel haustra and dilated colonic segments might be visualized [234]. Other diagnostic tests such as colonoscopy and anorectal manometry have been used. However, they have been unable to yield clinically relevant information and thus are not recommended for evaluating megacolon. This condition is defined as a sigmoid or descending colon with a diameter >6.5 cm, an ascending colon with a diameter > 8 cm, or a cecum > 12 cm in diameter (Figure 6) [234]. Since dilation may also occur in a longitudinal dimension, the term dolichocolon has been created to define a length greater than 70 cm from the splenic flexure to the anus [234]. A staging system has been proposed evaluating for key radiographic characteristics (Table 4).

Management of chagasic megacolon is usually done through lifestyle modifications that improve colonic transit, such as high-fiber diets, high water intake, and exercise. Pharmacological agents such as osmotic laxatives are also used. Surgical management is only considered in cases of chronic refractory constipation (1 to 2 bowel movements per month, referred to as colonic inertia) or in the case of important complication such as volvulus, stercoral ulcer, or recurrent bacterial infection requiring in-hospital treatment [235].

### 5.9. Chagas Disease and Gastrointestinal Cancer

It has long been hypothesized that CD involvement of the esophagus, stomach, and colon was associated with cancer development [236,237]. Thorough research has been done and a clear association has been found regarding chagasic megaesophagus and the development of esophageal cancer [236,238,239]. Between 3.9% up to 10% of patients with chagasic megaesophagus develop esophageal cancer related to their underlying disease, conceding them a risk 33 times greater than healthy subjects [238,240,241]. Most of these neoplasms are squamous cell carcinomas (88%), while the minority of them are adenocarcinomas (12%) [236]. 

GI CD involvement has also been associated with an increased risk of developing gastric carcinoma [242,243]. While this observation has been frequent, the pathophysiology behind this presents itself as a complex process given the fact that a higher prevalence of Helicobacter pylori infection is also seen in patients with CD, both in rural and non-rural populations [236,244,245,246]. Moreover, this finding has been revisited and also, a higher frequency of peptic ulcers has been observed in patients with CD when compared with healthy controls [244,247]. While the complex nature of the relationship between CD and the infection of H. pylori remains to be discovered, a clear association exists between CD and an increased risk of developing gastric carcinoma.

Finally, despite equivocal initial reports and associations drawn upon a relationship between CD and an increased risk of colon cancer [237], evidence has rejected a causal association [248,249]. The analysis of over 4690 necropsies and about 24,209 surgical pathology specimens showed no differences between chagasic and non-chagasic tissue as for the prevalence of malignant colonic lesions [250]. Nevertheless, it remains an area of early research, and molecular models are still under analysis to better understand the effects and pathophysiology behind chagasic megacolon [229,251,252].

## 6. Central Nervous System Involvement

Rarely, CD presents with neurological involvement. Although uncommon, both the acute and chronic presentations of CD in the nervous system usually entail high morbidity and mortality [253]. In the acute form, CD most often affects children under 2–3 years of age and presents with a clinical picture similar to that of a viral or bacterial meningoencephalitis, including seizures, headache, confusion, irritability, vomiting, and hypertonia [254]. On occasions, focal deficits might be seen. The estimated frequency of this type of presentation is around 0.8% [253]. Curiously, when there is central nervous system involvement, it usually remains the unique organ system involved in up to 60% of patients. However, a clear association has also been seen in patients with severe acute cardiac forms, in which a high level of parasitemia correlates with the severity of the disease; in these patients, *T. cruzi* has also been found in cerebrospinal fluid samples [253,255,256]. In these patients, histopathological analysis of the cerebral tissue demonstrates encephalitis with foci of glial and microglial granuloma-like nodules called chagomas. Also, amastigotes can be identified in microglial cells. It remains unknown whether the parasite directly invades neurons or if damage to these cells is only immune-mediated and secondary to reactive gliosis [254]. 

Despite extensive research on chronic alterations in the nervous system secondary to the chronic phase of *T. cruzi’s* infection, there is a lack of evidence suggesting a causal relationship [257]. Most histopathological studies agree that the changes seen in the brain caused mainly by residual anatomical changes following the acute phase [256,258]. Moreover, the array of symptoms is unspecific and lacks uniformity amongst different reports, without definite evidence of organic sequelae secondary to the severe acute form with profound immune-mediated damage [253,257]. 

In patients presenting with an acute central nervous system involvement, a lumbar puncture should be performed as well as the routine diagnostic tests already mentioned for detection of *T. cruzi* infection [253,255]. Cerebrospinal fluid analysis might reveal increased white blood cell count, predominantly with lymphocytes and increased protein levels. Centrifugation might reveal trypomastigotes [255]. Although not necessarily required, neuroimaging might reveal inflammatory changes, and, in severe cases, calcifications in periventricular areas. Chagomas are mostly observed in immunosuppressed patients with an acute form that follows reactivation [251].

Treatment with anti-trypanosomal agents like benznidazole or nifurtimox is mandatory at doses of 5–7 mg/kg and 8–10 mg/kg, respectively, for 60 to 90 days, depending on clinical judgment [253,259]. Despite the lack of studies evaluating the penetrance of these drugs through the brain-blood barrier, improvement in symptoms is usually seen after 2 to 3 days of treatment. In severe cases of meningoencephalitis, higher doses might be required. 

## 7. Conclusions

Chagas disease remains a highly prevalent disease with a profound impact in morbidity and mortality in endemic and non-endemic countries. Chronic complications of CD occur in a highly complex tripartite host-vector-pathogen interaction environment in which vector endemicity, genetic factors of the parasite, and immune regulations create a clinically variable phenotype which may involve several organ systems in different degrees of severity. An interdisciplinary patient-centered approach is needed in order to adequately address the multiple clinical features present in chronic CD and, in particular, those with a higher degree of impact in the patient’s quality of life. Furthermore, future research efforts should be focused towards controlling vector reservoirs, untangling the complex pathophysiologic phenomena of chronic complications, promoting prompt diagnosis and secondary prevention programs, and identifying as well as targeting potential therapeutic molecules with higher effectiveness and safety profiles than the currently available treatment regimes.

## Figures and Tables

**Table 1:** CD estimated prevalence in Latin and Central American countries and the United States of America based on several sources.

Country	Estimated Prevalence (in %)			
	WHO [66]	PAHO [67]	Guerri-Gutenberg et al. [68]	Schmunis et al. [69]
Argentina	3.61	4.13	4.90	8.20
Belize	-	0.74	-	-
Bolivia	6.10	6.75	14.80	15.40
Brazil	0.61	1.02	0.80	1.30
Chile	0.7	0.99	1.20	2.80
Colombia	0.95	0.96	1.20	0.48
Costa Rica	0.2	0.53	11.70	4.30
Ecuador	1.38	1.74	0.20	1.20
El Salvador	1.3	3.37	1.50	6.10
Guatemala	1.2	1.98	-	7.89
Guyana	-	1.29	-	-
Honduras	0.9	3.05	-	5.80
Mexico	0.78	1.03	0.50 to 6.80	0.70
Nicaragua	0.5	1.14	-	1.70
Panama	0.5	0.006	-	9.02
Paraguay	2.1	0.69	4.50	9.30
Peru	0.4	2.54	0.20	3.00
Suriname	-	-	-	0.10
Uruguay	0.2	0.66	0.60	1.20
USA	0.089 to 0.1 *			
Venezuela	0.7	1.16	1.30	4.00

* Estimated from: [70,71].

**Table 2:** Stages of Ch-CMP based on current guidelines.

A	B	C	D
Patients without symptoms of overt heart failure who are at risk of developing Ch-CMP. No structural evidence of cardiac disease.	Asymptomatic patients: B1: Mild structural changes B2: Decreased LVEF	Patients with heart failure symptoms due to severely decreased LVEF	Patients with heart failure refractory to therapy and in need of advanced interventions

Ch-CMP: Chagas cardiomyopathy. LVEF: Left ventricle ejection fraction. Adapted from Pino-Marín et al. [113].

**Table 3:** Severity staging scale for the evaluation of chagasic megaesophagus.

Stage	Description
I	Normal esophageal diameter with minimal contrast retention.
II	Moderate esophageal dilation with mild contrast retention and moderate uncoordinated activity of the lower esophageal sphincter.
III	Large increase in esophageal diameter with an important contrast retention with low or absent esophageal motor activity.
IV	Profound, atonic dilation of the esophagus with a large increase in volume. The esophagus lies on the right diaphragmatic dome.

**Table 4:** Radiographic staging system for chagasic megacolon.

Stage	Radiographic Description
0	No apparent alterations on barium enema.
I	Dolichocolon: longitudinal dilation >70 cm from the splenic flexure to the anus.
II	Dolichomegacolon: longitudinal dilation >70 cm from the splenic flexure to the anus; AND a transverse diameter at the sigmoid or descending colon >6.5 cm, >8 cm at the ascending colon, OR > 12 cm at the cecum.

## Data Availability

No new data were created or analyzed in this study. Data sharing is not applicable to this article.

## Abbreviations

ACE-I	Angiotensin-converting enzyme inhibitor
ARB	Angiotensin II receptor blocker
ARNI	Dual angiotensin receptor and neprilysin inhibitor
CD	Chagas disease
Ch-CMP	Chagasic cardiomyopathy
CINE	Steady state free precession sequence
CMR	Cardiac magnetic resonance
CRIT	C2 receptor inhibitor trispanning protein
CRP	Complement regulatory protein
DALY	Disability-adjusted life-years
DGF	Dispersed gene family proteins
DTU	Discrete typing unit
ER	Endoplasmic reticulum
FESOD	Iron superoxide dismutases
GI	Gastrointestinal
INF	Interferon
LGE	Late gadolinium enhancement
LVEF	Left ventricle ejection fraction
MASP	Mucin associated surface proteins
MHC	Major histocompatibility complex
MRA	Mineralocorticoid receptor antagonist
NYHA	New York Heart Association
PCR	Polymerase chain reaction
PR	Proline racemase
RBBB	Right-bundle branch block
RHS	Retrotransposon hot spot
SCD	Sudden cardiac death
SGLT-2	Sodium-glucose transporter 2
TcCRT	Trypanosoma cruzi calreticulin
TcGP	Trypanosoma cruzi glutathione peroxidase
TcPRACA	Trypanosoma cruzi proline racemase A
TcPRACB	Trypanosoma cruzi proline racemase B
T-DAF	Trypomastigote decay accelerating factor
TSSA	Trypomastigote small surface antigen
TTE	Transthoracic echocardiogram
